# Alagille Syndrome Mimicking Biliary Atresia in Early Infancy

**DOI:** 10.1371/journal.pone.0143939

**Published:** 2015-11-30

**Authors:** Tomáš Dědič, Milan Jirsa, Radan Keil, Michal Rygl, Jiri Šnajdauf, Radana Kotalová

**Affiliations:** 1 Department of Paediatrics, 2nd Faculty of Medicine, Charles University in Prague and Motol University Hospital, Prague, Czech Republic; 2 Laboratory of Experimental Hepatology, Institute for Clinical and Experimental Medicine, Prague, Czech Republic; 3 Department of Internal Medicine, 2nd Faculty of Medicine, Charles University in Prague and Motol University Hospital, Prague, Czech Republic; 4 Department of Paediatric Surgery, 2nd Faculty of Medicine, Charles University in Prague and Motol University Hospital, Prague, Czech Republic; Texas A&M Health Science Center, UNITED STATES

## Abstract

Alagille syndrome may mimic biliary atresia in early infancy. Since mutations in *JAG1* typical for Alagille syndrome type 1 have also been found in biliary atresia, we aimed to identify *JAG1* mutations in newborns with proven biliary atresia (n = 72). Five biliary atresia patients with cholestasis, one additional characteristic feature of Alagille syndrome and ambiguous liver histology were single heterozygotes for nonsense or frameshift mutations in *JAG1*. No mutations were found in the remaining 67 patients. All “biliary atresia” carriers of *JAG1* null mutations developed typical Alagille syndrome at the age of three years. Our data do not support association of biliary atresia with *JAG1* mutations, at least in Czech patients. Rapid testing for *JAG1* mutations could prevent misdiagnosis of Alagille syndrome in early infancy and improve their outcome.

## Introduction

Biliary atresia represents a frequent cause of neonatal cholestasis. The disease is defined as occlusive panductular cholangiopathy affecting both intra- and extrahepatic bile ducts [1]. Aetiology of biliary atresia is still unknown; genetic background might play a role in some cases [2–5]. Rapid diagnosis and therapy of biliary atresia is essential because the efficiency of surgical reconstruction of the extrahepatic biliary tract by portoenterostomy (the Kasai procedure) is limited by the age of the patient [6]. Most patients require liver transplantation at later age.

Alagille syndrome (OMIM #118450), a complex multisystem autosomal dominant disorder with incomplete penetrance, may be indistingushible from biliary atresia in the neonatal period. The disease is defined clinically by the association of at least three of the five major features—chronic cholestasis, congenital heart disease, skeletal anomalies (typically butterfly vertebrae), ocular abnormalities (primarily posterior embryotoxon) and peculiar face—and genetically by heterozygous state for mutations in *JAG1* [7, 8] or rarely in *NOTCH2* [9]. Mutations in *JAG1* have also been found in a subset of patients with biliary atresia [4]; however, their contribution to clinical symptoms and the disease course remains unclear. Moreover, some patients with Alagille syndrome are likely misdiagnosed as biliary atresia due to variable expression of clinical features of the syndrome in early infancy, which may result in minimal or subclinical disease that can be missed [10].

In this study we focused on patients with biliary atresia. Our aim was to identify and characterize individuals carrying *JAG1* mutations among 72 patients with biliary atresia and to confirm the diagnosis in 4 patients with suspected Alagille syndrome having intrahepatic cholestasis without biliary atresia.

## Materials and Methods

### Subjects

Three hundred ten children with neonatal cholestasis were hospitalized at the Department of Paediatrics, Motol University Hospital, Prague, between January 1998 and January 2012. Endoscopic retrograde cholangiopancreatography was performed in 127 of these patients and the findings indicated presence of biliary atresia in 96 patients. The diagnosis was confirmed at laparotomy, with intraoperative cholangiography when necessary, and by histological examination of wedge liver biopsy specimens. Twenty four patients died before the material for genetic testing could be obtained. Mutational analysis of *JAG1* was performed in the remaining 72 individuals with biliary atresia treated by the Kasai procedure and in 4 patients (3 unrelated index subjects plus one symptomatic sibling) with suspected Alagille syndrome with intrahepatic cholestasis but normal extrahepatic biliary tree. All examined patients were central Europeans of Czech origin.

The patient studies were performed in accordance with the ethical principles outlined in the Declaration of Helsinki and approved by the Institutional Review Board of the Faculty Hospital Motol and the 2^nd^ Faculty of Medicine, Charles University in Prague. Written informed consent was obtained from parents on behalf of all children enrolled and from all adult participants included in the study.

### Mutation analysis

Genomic DNA was extracted using a Qiagen DNA micro kit (QIAgen, Hilden, Germany) according to the manufacturer´s specifications. *JAG1* was analysed by direct sequencing of genomic DNA. All 26 coding exons were amplified by PCR using the intronic primer pairs presented in the S1 Table. Some of the amplified fragments were gel-purified and extracted from the 1% agarose gel using the QIAquick Gel Extraction Kit (QIAgen, Hilden, Germany). Sequence analysis with the Big Dye Terminator v.3.1 Cycle Sequencing Kit (Applied Biosystems, Foster City, CA) was performed on the ABI PRISM 3130 Genetic Analyzer (Applied Biosystems). The data were analysed using the Applied Biosystems SeqScape software. The sequences were compared with the NCBI GenBank reference sequences NG_007496.1 and NM_000214.2 for genomic DNA and mRNA, respectively.

PCR products with difficult to read sequences due to heterozygous deletions were cloned into a pCR4.1-TOPO plasmid vector (Invitrogen, Carlsbad, CA), and the wild-type and mutated alleles were sequenced separately.

In case of no pathogenic mutation detection by Sanger sequencing, multiplex ligation-dependent probe amplification (MLPA) dosage analysis was carried out to look for partial or whole *JAG1* gene deletions. MLPA analysis was carried out according to the manufacturer´s instructions using the P184 MLPA kit available from MRC-Holland (Amsterdam, The Netherlands). Mutational analysis was also run in parents and siblings of index patients carrying mutations in *JAG1*.

Pathogenicity of amino acid substitutions was evaluated with the PredictSNP 1.0 consensus classifier for prediction of disease-related mutations [11]. Frequencies of variant alleles were compared with those found in NCBI GenBank SNP database, 1000 Genomes project, Exome Aggregation Consortium database and Exome Sequencing Project.

## Results

Five null mutations in *JAG1* have been detected in five unrelated patients presenting initially as biliary atresia (patients 1–5 in Table 1). Four of them are novel whereas the fifth (p.Tyr320*, Human Gene Mutation Database accession No. CM124879) has been reported recently [12]. Only one of the mutations (p.Cys438Serfs found in patient 4) was inherited from the patient´s mother characterized by craniofacial dysmorphia. The remaining four mutations are of *de novo* origin. All five mutation carriers are single heterozygotes.

**Table 1:** Mutations in *JAG1* found in patients with biliary atresia and Alagille syndrome.

Patient	Sex	Diagnosis	Exon	Mutation	Protein	Mutation type	Mutation origin
1	M	BA	2	**c.327_330delCAAG**	**p.Lys110Profs** * **50**	frameshift	*de novo*
2	M	BA	6	**c.879_880delTG**	**p.Cys293** *	nonsense	*de novo*
3	F	BA	7	c.960T>A	p.Tyr320*	nonsense	*de novo*
4	F	BA	10	**c.1313_1314delGT**	**p.Cys438Serfs** * **10**	frameshift	maternal
5	M	BA	15	**c.1899_1900delTG**	**p.Cys633** *	nonsense	*de novo*
6	M	AGS	3	**c.402G>T**	**p.Leu134Phe**	missense	maternal
7*	M	AGS	16	**c.2050delG**	**p.Asp684Thrfs** * **59**	frameshift	maternal
8*	M	AGS	16	**c.2050delG**	**p.Asp684Thrfs** * **59**	frameshift	maternal
9	F	AGS	23	**c.2913_2914delAC**	**p.Pro972Argfs** * **10**	frameshift	maternal

* siblings;

AGS, Alagille syndrome; BA, biliary atresia; novel mutations are in **bold**.

Even after careful re-examination of these five “biliary atresia”patients prompted by their genetic findings, none of them met the diagnostic criteria for Alagille syndrome at time of hospitalization for neonatal cholestasis. All had systolic murmur and one patient had *foramen ovale apertum* but neither structural anomalies of the heart nor stenosis of branches of pulmonary artery could be documented (Table 2). Moreover, in wedge liver biopsy specimens obtained during the Kasai surgery, portal expansion with ductular proliferation and occasional bile ducts but no bile duct paucity was observed. On long-term follow-up, all five patients developed clinical signs sufficient for clinical diagnosis of Alagille syndrome before 3 years of age (Table 2). Three patients were enlisted for liver transplantation with the end stage liver disease at 3 years of age. Two of them (patients No. 1 and 5 in Tables 1 and 2) underwent successful liver transplantation whereas the third patient (No. 4) died from variceal bleeding.

**Table 2:** Clinical features present in carriers of *JAG1* mutations at the time of hospitalization for neonatal cholestasis and at 3 years of age.

Patient	ERCP	Cholestasis	Heart disease	Embryotoxon posterior	Skeletal anomalies	Peculiar face	AGS criteria
1	BA type 3	+	+^#^	-	-	+^#^	1; 3
2	BA type 3	+	+^#^	-	+	+^#^	2; 4
3	BA type 3	+	+^#^	+	-	-	2; 3
4	BA type 3	+	+^#^	+^#^	+	+^#^	2; 5
5	BA type 4	+	+^#^	maculopathy^#^	+	+^#^	2; 5
6	normal	+	+	microphtalmy	-	+	4; 4
7*	normal	+	+	-	+	+	4; 4
8*	not done	+	*+*	+	-	+	4; 4
9	normal	+	+	+	+	+	5; 5

* siblings;

ERCP, endoscopic retrograde cholangiopancreatography; BA, biliary atresia; BA type 3 –gallbladder, cystic duct and common bile duct are patent; BA type 4 –atresia of all the extrahepatic bile ducts; clinical features were present (+) or missing (-),

^#^ indicates clinical features not present at the age of 2 months;

AGS criteria indicate the number of major clinical features (diagnostic criteria) of Alagille syndrome present at the age of 2 months and 3 years, respectively.

No predictably pathogenic mutations in *JAG1* have been detected in the remaining 67 patients with biliary atresia by Sanger sequencing and the multiplex ligation-dependent probe amplification assay (S2 Table).

**Fig 1:**
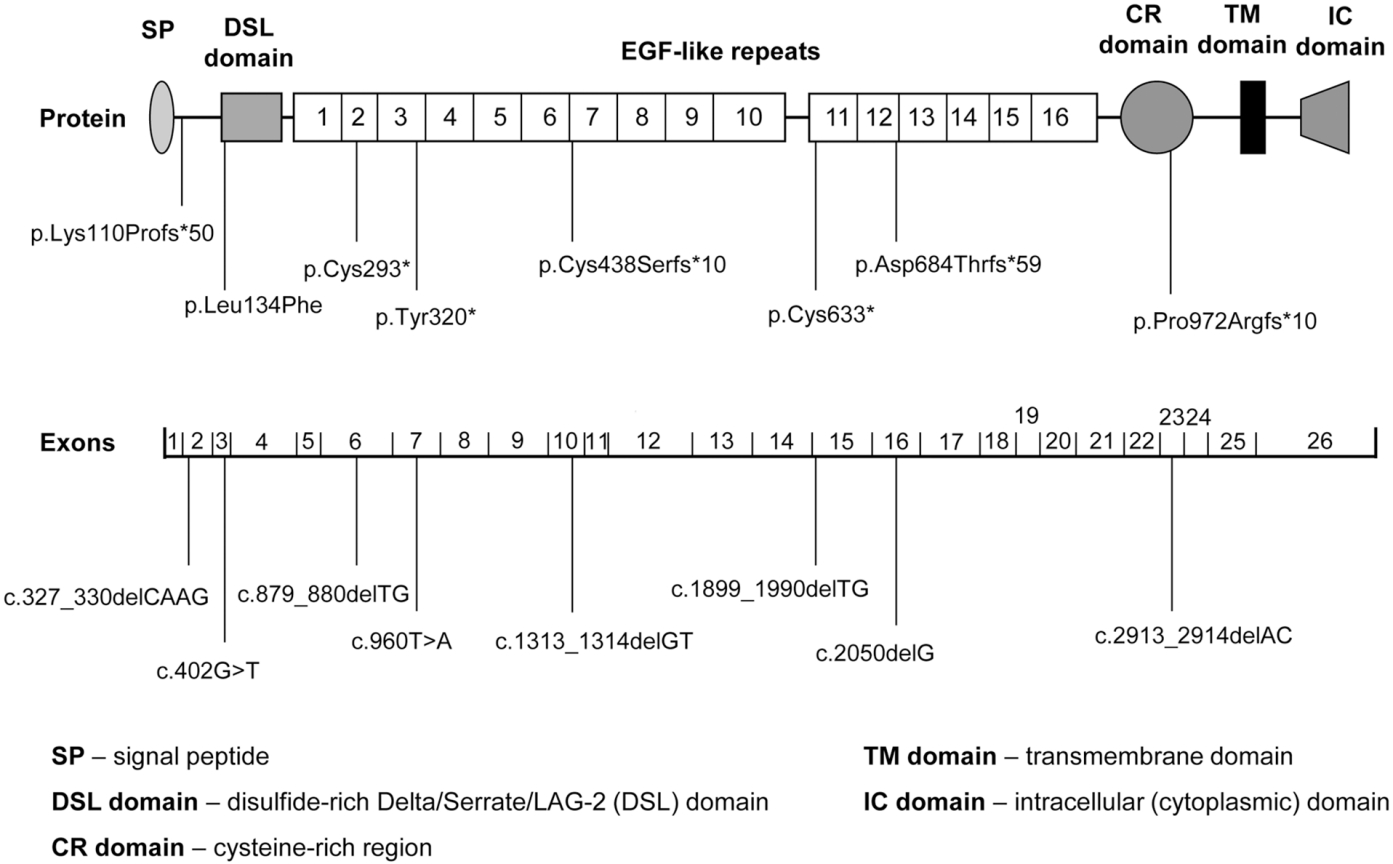
Schematic representation of the Jagged1 protein and spliced *JAG1* mRNA with mutations found in our patients.

In contrast, three novel mutations were found in three index subjects and one sibling with typical features of Alagille syndrome at the age of six months (patients 6–9 in Table 2). All mutations present in heterozygous state are of maternal origin. The otherwise healthy mothers had craniofacial dysmorphic features. The two frameshift mutations create early stop codons and, provided that the mutated mRNA is not eliminated by nonsense-mediated decay, result in non-functional truncated proteins lacking the membrane and cytoplasmic domain (Fig 1). The missense mutation c.402G>T (p.Leu134Phe) affects exon 3, which encodes a part of the DSL domain of *JAG1* (Fig 1). The domain is highly conserved between different species and is essential to activate Notch receptors [13]. The substitution of 134Leu with phenylalanine is predicted as pathogenic using the PredictSNP 1.0 classifier, the reliability of the prediction is 87% (S3 Table). The frameshift mutation p.Asp684fs found in patient 2 is also present in his symptomatic brother (patient 8 in Table 2). Both siblings differ in their clinical presentation (see Table 2); however, this is well in line with the known highly variable expressivity of the disease [14].

Location in the spliced mRNA and protein sequence of all 8 mutations we have found in our patients is depicted in Fig 1. Positions of the nonsense and frameshift mutations indicate the exact or approximate ends of the hypothetical truncated non-functional proteins. Pathogenicity of the mutations is further supported by their unique character: None of them has occurred in the GenBank SNP database, 1000 Genomes project, Exome Aggregation Consortium database and the Exome Sequencing Project (S3 Table).

## Discussion

Confirmation of the clinical diagnosis of Alagille syndrome in patients without biliary atresia by finding mostly novel pathogenic mutations in *JAG1* was not surprising. In contrast, the role of *JAG1* mutations in aetiology of biliary atresia is still unclear. Since defective Jagged1/Notch-2 signalling is responsible for transdifferentiation of hepatoblasts to biliary epithelia [15] and mutations in either *JAG1* or *NOTCH2* are known to cause Alagille syndrome type 1 and 2, respectively, association of *JAG1* with biliary atresia reported by Kohsaka et al. [4] would be acceptable. These authors found 9 missense mutations present in 11 out of 102 patients with biliary atresia where 28 of them underwent liver transplantation before 5 years of age. None of the mutation carriers developed typical clinical features of Alagille syndrome before the age of 5 years. According to the first hypothesis presented in [4] patients with biliary atresia and *JAG1* deficiency could represent atypical Alagille syndrome with not fully expressed clinical features of Alagille syndrome. Alternatively, mutated Jagged1 protein may affect inflammatory processes in the liver via the Jagged1/Notch-2 pathway-mediated regulation of cytokine expression.

Neither we in this study nor any other group to the best of our knowledge, observed a carrier of *JAG1* mutation with biliary atresia and no clinical features of Alagille syndrome similar to the 11 carriers of missense mutations reported by the Kohsaka´s group [4]. Association of these missense mutations with Alagille syndrome has not been reported as well. Such discrepancy may be attributed to differences in ethnicity of biliary atresia patients studied in Japan, Europe and USA. Likewise, the role of the *JAG1* mutations in genetic aetiology of biliary atresia may be questioned. Eight of the 9 mutations reported by Kohsaka et al. [4] were identified in sporadic cases. Since *JAG1* is not a strong biliary atresia candidate disease gene concurrent with the sporadic incidence of the disease, the reported occurrence of rare *JAG1* missense mutations does not univocally prove the genotype—phenotype correlation. The correlation is further doubted by the fact that pathogenicity prediction of all but two mutations reported in [4] has been evaluated *in silico* as neutral by several prediction programs exploited by the PredictSNP 1.0 classifier (S3 Table) and the predictably pathogenic mutation p.Pro871Arg (dbSNP rs35761929) is classified as benign in the ClinVar database owing to its high frequency in the 1000 Genomes project, Exome Aggregation Consortium database and the Exome Sequencing Project (S3 Table).

In our cohort of 72 patients with biliary atresia of which 27 were enlisted for liver transplantation before 5 years of age, we found 5 carriers of *JAG1* null mutations but no carrier of a single unique potentially pathogenic missense mutation. All these patients who initially presented as biliary atresia developed sufficient number of clinical signs typical for Alagille syndrome before 3 years of age.

One of the main diagnostic features of Alagille syndrome is bile duct paucity, which is more common later in infancy and childhood [16–18]. Ductular proliferation is present in a small number of infants with Alagille syndrome, leading to significant diagnostic confusion. Because of the variability in the early histopathology of the liver in Alagille syndrome, a number of patients have been misdiagnosed as having biliary atresia [16, 19, 20]. Surgical reconstruction of extrahepatic bile duct system in patients with Alagille syndrome does not correct for the loss of the bile ducts within the liver and liver transplantation is preferred. This is a reason why early molecular diagnostics could be considered in selected cases of overlapping Alagille syndrome and biliary atresia.

A question arises about how the candidates for accelerated *JAG1* sequencing should be selected. None of our 5 patients with mutations in *JAG1* presenting as biliary atresia in early infancy met the diagnostic criteria for Alagille syndrome at the critical time of hospitalisation for neonatal cholestasis. However, all had pathological findings on endoscopic retrograde cholangiopancreatography (see Table 2) and systolic murmur but no signs of pulmonary stenosis on echocardiography. Therefore we suppose that cardiological phenotype (typical pulmonary stenosis) can develop quite slowly. Since craniofacial dysmorfia and embryotoxon posterior are difficult to find in newborns or patients in early infancy and liver histology may be inconclusive, we suggest that patients with neonatal cholestasis due to biliary atresia and either systolic murmur or any single clinical feature characteristic for Alagille syndrome should be elicited for mutational analysis of *JAG1* before surgery.

In conclusion, our findings do not support association of biliary atresia with *JAG1* mutations. In addition to liver histology, mutational analysis of *JAG1* could be useful for diagnosis of the “grey zone”patients with Alagille syndrome presenting initially as biliary atresia in early infancy.

## Supporting Information

S1 Table Primer pairs used for amplification of the *JAG1* coding regions. (DOC)

S2 Table Common variants detected in protein coding regions and splice sites of *JAG1* in patients with biliary atresia and Alagille syndrome. (XLSX)

S3 Table Population frequency data and pathogenicity predictions for mutations found in our study and for those reported by Kohsaka et al.[4]. (XLSX)
