# Supplementary material for: Alagille Syndrome Mimicking Biliary Atresia in Early Infancy
Source: PLoS One. 2015 Nov 30;10(11):e0143939. doi: 10.1371/journal.pone.0143939 (PMC4664419; doi:10.1371/journal.pone.0143939)
Supplement: S1 Table — (DOC) [file pone.0143939.s001.doc]

**S1 Table:** Primer pairs used for amplification of the *JAG1* coding regions.

| **Pair** | **Forward primer** | **Reverse primer** | **Exon(s)** |
| --- | --- | --- | --- |
| 1 | 5´-agtcgtgcatgctccaatc-3´ | 5´-acgagtgcggaagaaatcc-3´ | 1 |
| 2 | 5´-agggagtcgccacctctatac-3´ | 5´-caagccaaagccctttaaatc-3´ | 2 |
| 3 | 5´-gagtcatttgtcaacctgggaag-3´ | 5´-agggaatgagttaactgggattctg -3´ | 3 |
| 4 | 5´-aagccaaaatggtcagggaag-3´ | 5´-atcccaccccacctgagatag-3´ | 4 |
| 5 | 5´-aaaatcgtggaatttgcagac-3´ | 5´-aaagctttcttgttctccaagg-3´ | 5 |
| 6 | 5´-agcctggactttgcagtcttc-3´ | 5´-acccacacagcattcaaggag-3´ | 6 |
| 7 | 5´-ggtgggatggcactaatttgg-3´ | 5´-tcgctgagctgtctctaaccac-3´ | 7 |
| 8 | 5´-gtgtgggattcggttggag-3´ | 5´-accgagacattcacactggac-3´ | 8 |
| 9 | 5´-ggtgtctggctcttcaatgac-3´ | 5´-ctcgtcttctgtaatggctttg-3´ | 9 |
| 10 | 5´-cagacaaactctggcctgttc-3´ | 5´-agcaagtcggctacccaag-3´ | 10 |
| 11 | 5´-atctcacggaaagcacacagg-3´ | 5´-cacgaggctggggtaacataag-3´ | 11 |
| 12 | 5´-aaccagctaaaccgcaacag-3´ | 5´-ttccagacacaagagctgagg-3´ | 12 |
| 13 | 5´-ccagagaagttatcgtgacacc-3´ | 5´-aacaaggggcagtggtagtaag-3´ | 13 |
| 14 | 5´-cttaggaatgccgcatctg-3´ | 5´-actctagcctgggcaacaac-3´ | 14 |
| 15 | 5´-caggtgcattgtgtcaggag-3´ | 5´-aaatcactgcggtcttgcttc-3´ | 15 |
| 16 | 5´-tcatttccatgggacttccag-3´ | 5´-cgtggcctcatcacactgac-3´ | 16 |
| 17 | 5´-tggtagttggccttggatgc-3´ | 5´-cgaccaccctccctgagtatc-3´ | 17 |
| 18 | 5´-tgcccctccagactgtttc-3´ | 5´-aagtccccaagggtgtcag-3´ | 18 |
| 19 | 5´-tcatttgggtattaggaaaataagtgg -3´ | 5´-agtctgaggcccagaacttgc -3´ | 19 |
| 20 | 5´-tggtgacatgtgagtgattgg-3´ | 5´-gatgaaagtcttggggtgagg-3´ | 20 |
| 21 | 5´-caatctcaaaacattgccacac-3´ | 5´-gtcaaatggtgactgcaaagc-3´ | 21 |
| 22 | 5´-cagcaaaggcaggaagtacac-3´ | 5´-agatcctagctcatggcattc-3´ | 22, 23 |
| 23 | 5´-tgaacttagccagcctcaaag-3´ | 5´-actgccagataatccctcgac-3´ | 24, 25 |
| 24 | 5´-gataaagggcaggagaaccac-3´ | 5´-cagggattctaagtcagcaacg-3´ | 26 |
